# Zika Virus Persistently and Productively Infects Primary Adult Sensory Neurons In Vitro

**DOI:** 10.3390/pathogens6040049

**Published:** 2017-10-13

**Authors:** Brianna K. Swartwout, Marta G. Zlotnick, Ashley E. Saver, Caroline M. McKenna, Andrea S. Bertke

**Affiliations:** 1 Translational Biology, Medicine and Health, Virginia Tech Carilion Research Institute, Virginia Tech, Roanoke, VA 24016, USA; bkswart@vt.edu; 2 Virginia-Maryland College of Veterinary Medicine, Blacksburg, VA 224061, USA; marta1@vt.edu (M.G.Z.); aes27@vt.edu (A.E.S.); 3 School of Neuroscience, Virginia Tech, Blacksburg, VA 24061, USA; mckennac@vt.edu; 4 Population Health Sciences, Virginia-Maryland College of Veterinary Medicine, Blacksburg, VA 24061, USA

**Keywords:** Zika virus, sensory, autonomic, neurons, Guillain-Barré, satellite glial cells

## Abstract

Zika virus (ZIKV) has recently surged in human populations, causing an increase in congenital and Guillain-Barré syndromes. While sexual transmission and presence of ZIKV in urine, semen, vaginal secretions, and saliva have been established, the origin of persistent virus shedding into biological secretions is not clear. Using a primary adult murine neuronal culture model, we have determined that ZIKV persistently and productively infects sensory neurons of the trigeminal and dorsal root ganglia, which innervate glands and mucosa of the face and the genitourinary tract, respectively, without apparent injury. Autonomic neurons that innervate these regions are not permissive for infection. However, productive ZIKV infection of satellite glial cells that surround and support sensory and autonomic neurons in peripheral ganglia results in their destruction. Persistent infection of sensory neurons, without affecting their viability, provides a potential reservoir for viral shedding in biological secretions for extended periods of time after infection. Furthermore, viral destruction of satellite glial cells may contribute to the development of Guillain-Barré Syndrome via an alternative mechanism to the established autoimmune response.

## 1. Introduction

Zika virus (ZIKV) is an emerging pathogen of global health concern due to its connection with microcephaly and Guillain-Barré Syndrome (GBS). Historically, ZIKV presented as subclinical or mild illness with fever, headache, and diffuse joint pain [1]. Recent epidemics outside of Africa on the Micronesian island Yap, French Polynesia, and Brazil have been associated with increased microcephaly, blindness, and neurological disorders in infants born to women infected while pregnant. In adults, approximately 20% of those infected develop symptoms, which are typically self-limiting [2]. However, the concerns regarding the connection between GBS and ZIKV infection did not emerge until the French Polynesian epidemic. Subsequently, cases of meningoencephalitis and myelitis have also been reported following ZIKV infection [3]. In a case-control study, French scientists describing the correlation between ZIKV and GBS in French Polynesia showed that 1 in every 4200 people with ZIKV suffered from GBS, as compared to the global incidence of 1 in 100,000 people per year [4]. GBS is characterized by numbness and tingling in the extremities, weakness in limbs, and sometimes paralysis. Symptoms of GBS are often transient, rarely resulting in long-term consequences, but severe cases can last for months and lead to respiratory failure requiring extended stays in intensive care on mechanical ventilation [5].

ZIKV belongs to the Flaviviridae family, along with other clinically notable viruses, such as dengue, Japanese encephalitis, and West Nile viruses, all of which are capable of causing encephalitis and other neurological disorders. The prototype ZIKV strain, MR766, was isolated from a Rhesus macaque in Uganda in 1947 and the first human strain, IbH30656, was isolated in Nigeria in 1952 [2]. ZIKV is transmitted by at least three species of Aedes mosquitoes [2,6,7], but unlike other neurotropic flaviviruses, ZIKV is also transmitted sexually [8,9]. Following ZIKV infection, the virus is shed in urine and semen for several months and has also been detected in vaginal secretions [10,11,12]. Although ZIKV infection of testes has been demonstrated in animal models [13,14,15,16], viral RNA was also present in seminal fluid in vasectomized mice, suggesting that prolonged ZIKV shedding in semen may originate from a persistent site of infection other than the testes [16]. Other sexually transmitted viruses, such as herpes simplex virus (HSV), are known to infect and establish latency in peripheral sensory and autonomic neurons that innervate the genitourinary tract and periodically shed viral progeny into semen and vaginal secretions [17,18,19,20]. Therefore, we sought to determine if the same neurons may also be a potential reservoir for persistent ZIKV. We determined that two African ZIKV strains, MR766 and IbH30656, persistently infect sensory neurons, but not autonomic neurons, that innervate the genitourinary tract. In addition, we found differences in neuronal infectivity between the two strains. Furthermore, both strains infect and kill satellite glial cells that surround and support peripheral neurons.

## 2. Results

**Figure 1:**
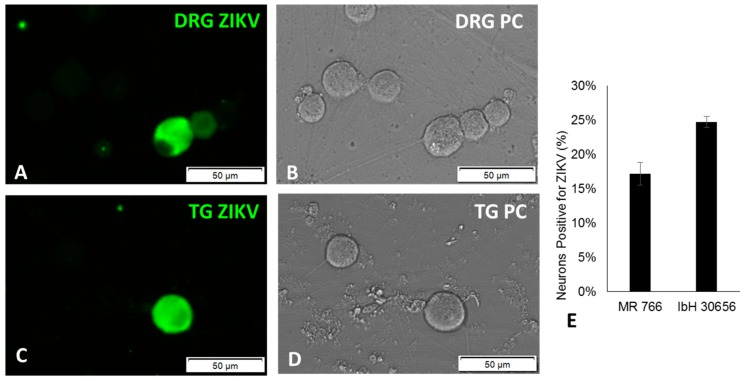
ZIKV productive infection in cultured primary adult peripheral sensory neurons, inoculated with 30 MOI ZIKV, 2 dpi: (**A**) dorsal root ganglion (DRG), immunofluorescence for ZIKV; (**B**) DRG, phase contrast (PC); (**C**) trigeminal ganglion (TG), immunofluorescence for ZIKV; (**D**) TG, phase contrast (PC); (**E**) percentage of DRG neurons positive for ZIKV antigen by immunofluorescence, 48 h post inoculation with 30 MOI ZIKV (*n* = 3 experiments in triplicate, SEM).

ZIKV infects sensory neurons of the trigeminal and dorsal root ganglia. To determine if ZIKV is able to infect peripheral sensory neurons that innervate the genitourinary tract, we isolated primary sensory neurons from the dorsal root ganglion (DRG) of six-week-old immunocompetent adult mice (outbred Swiss Webster) and inoculated the cultured neurons with a multiplicity of infection (MOI) of 30 PFU/cell of ZIKV strains MR766 and IbH30656. Forty-eight hours post inoculation (hpi), we detected ZIKV antigen in 17.22% of neurons inoculated with MR766 and 24.75% of neurons inoculated with IbH30656, using human anti-ZIKV serum (Figure 1A,B,E). Since ZIKV has also been detected in saliva and eyes [21,22,23,24,25,26], we also sought to determine if ZIKV infects sensory neurons of the trigeminal ganglion (TG), which innervates the mouth, nose, and eyes. Both MR766 and IbH30656 infected TG sensory neurons, similar to DRG neurons (Figure 1C,D).

**Figure 2:**
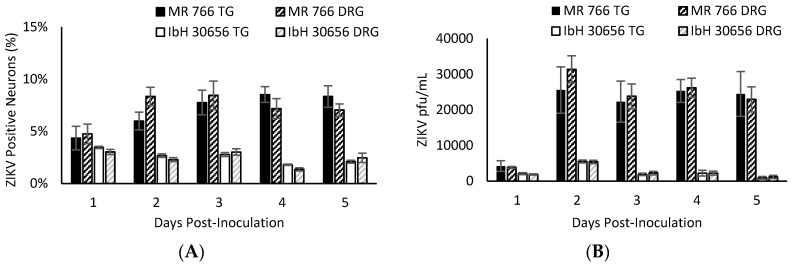
ZIKV productive infection of cultured primary adult sensory neurons isolated from TG and DRG of adult mice, following inoculation with 10 MOI ZIKV MR766 and IbH30656: (**A**) Percentage of ZIKV antigen positive sensory neurons, visualized by immunofluorescence with human anti-ZIKV serum over the course of five days; (**B**) Titers of infectious virus released into media over the course of five days, determined by plaque assay on Vero cells. (*n* = 6–10 independent experiments performed in duplicate, SEM).

ZIKV persistently infects sensory neurons. We observed that ZIKV infection did not appear to damage the infected adult sensory neurons, as these neurons retained axonal projections with no visual evidence of cellular or nuclear membrane disruption (Figure 1B,D). Therefore, we next infected primary adult sensory TG and DRG neuronal cultures with ZIKV and assessed the percentage of neurons infected over a period of five days. Different strains of ZIKV have been shown to have varying degrees of neurotropism in the central nervous system (CNS), so we sought to determine if the two different strains would infect different proportions of sensory neurons. At 24 hpi following inoculation of 10 MOI ZIKV, both MR766 and IbH30656 infected a similar percentage of sensory neurons in the TG or DRG cultures; 4.4% TG and 4.8% DRG neurons were positive for MR766, and 3.5% TG and 3.0% DRG neurons were positive for IbH30656 (Figure 2A). However, the percentage of TG neurons infected by MR766 significantly increased from 4.4% to 8.5% (*p* < 0.01) over the course of five days (Figure 2A). Similar percentages were detected for DRG neurons infected with MR766 (4.4% to 8.4%). In contrast, the percentage of sensory neurons infected by IbH30656 remained relatively constant, from 3.5% (TG) on Day 1 to 2.5% (TG) on Day 5 post inoculation (Figure 2A). Similar percentages were observed for DRG neurons infected with IbH30656 (3.0% to 2.1%). By 2 dpi, MR766 productively infected a significantly greater percentage of TG and DRG sensory neurons compared to IbH30656 (*p* = 0.003 and 0.0002, respectively). Thus, different strains of ZIKV from the same African lineage can persistently infect peripheral sensory neurons.

To establish that infection of peripheral sensory neurons can produce infectious viral progeny and potentially contribute to viral transmission at sites of innervation, we assessed the productivity of infection by performing titrations on media collected from infected TG and DRG neuronal cultures over the course of five days. MR766 and IbH30656 successfully replicated and released infectious progeny with peak production after two days in culture (Figure 2B). From 2 dpi going forward, infectious progeny release remained relatively constant, although MR766 released significantly greater numbers of infectious viral progeny (2.2 × 10^4^ to 2.6 × 10^4^ in TG and 2.1 × 10^4^ to 3.4 × 10^4^ in DRG neurons) over the course of the five day infection compared to IbH30656 (1.0 × 10^3^ to 5.5 × 10^3^ in TG and 1.1 × 10^3^ to 5.4 × 10^3^ in DRG neurons) (*p* < 0.01) (Figure 2B).

**Figure 3:**
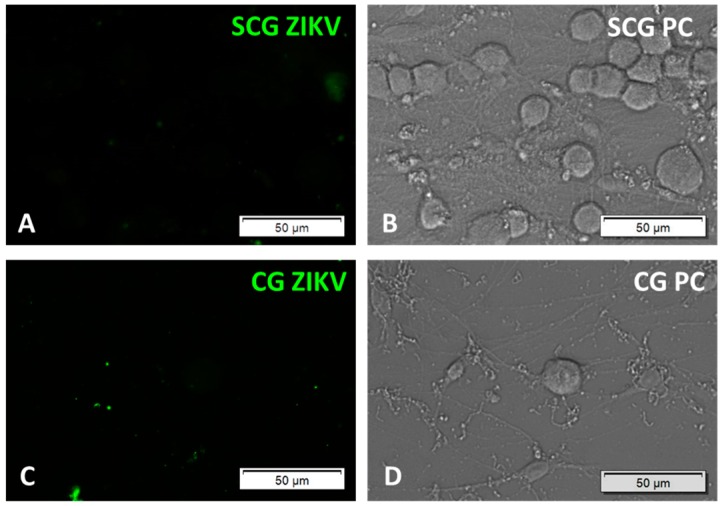
ZIKV productive infection in cultured primary adult peripheral autonomic neurons, inoculated with 10 MOI ZIKV, 2 dpi: (**A**) sympathetic superior cervical ganglion (SCG) neurons, immunofluorescence for ZIKV; (**B**) SCG, phase contrast (PC); (**C**) parasympathetic ciliary ganglion (CG) neurons, immunofluorescence for ZIKV; (**D**) CG, phase contrast (PC).

Autonomic neurons from peripheral ganglia are not permissive for ZIKV productive infection. The genitourinary tract and the facial mucosa and glands are extensively innervated by autonomic neuronal pathways, in addition to sensory neurons. To determine if autonomic neurons also support productive ZIKV infection, cultured primary adult murine sympathetic neurons isolated from the superior cervical ganglia (SCG), and parasympathetic neurons isolated from the ciliary ganglia (CG) were inoculated with MOI of 10 PFU/cell ZIKV strains MR766 and IbH30656. No evidence of ZIKV productive infection was identified by immunofluorescent staining (Figure 3).

**Figure 4:**
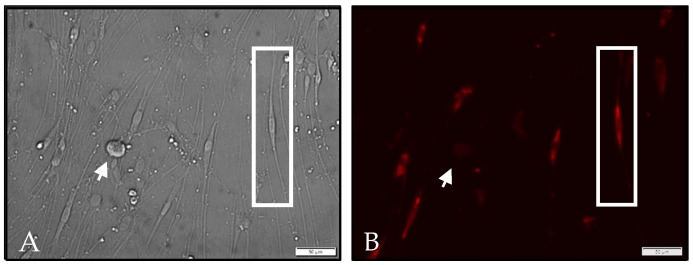
Satellite glial cells are permissive for ZIKV productive infection: (**A**) Phase contrast image showing several satellite glial cells near a single sympathetic neuron (white arrow); (**B**) Immunofluorescence image showing satellite glial cells positive for ZIKV antigen, while the sympathetic neuron remains negative (white arrow). The white rectangle highlights one representative satellite glial cell positive for ZIKV by immunofluorescence.

ZIKV infects and kills satellite glial cells. Satellite glial cells are an understudied type of glial cell that surround and protect the soma of ganglionic neurons in peripheral sensory and autonomic ganglia, and their role during the course of infection of the peripheral nervous system is unknown. Satellite glial cells are inherently present in our primary adult neuronal cultures, as these supporting cells tightly surround adult neurons and migrate from the surface of the neurons following dissociation and culture. Although no neurons were positive for ZIKV antigen in sympathetic neuronal cultures, the majority of the satellite glial cells were positive by immunofluorescence (Figure 4) in these cultures 24 hpi. Infection of satellite glial cells with ZIKV was terminal for the satellite glial cell population, as few intact satellite glial cells remained in the cultures 48–72 hpi, while the sympathetic neurons remained viable. The contribution of satellite glial cells to the pathology of ZIKV has yet to be discovered, but may be a key factor in the associated neurological complications.

## 3. Discussion

Our findings show that ZIKV productively and persistently infects a portion of trigeminal (TG) and dorsal root (DRG) sensory neurons, whereas autonomic sympathetic and parasympathetic neurons are not permissive for productive infection. Infection of TG sensory neurons, which innervate the mucosal surfaces and glands of the face, may contribute to ZIKV shedding at sites conceivably vulnerable to transmission. ZIKV has been detected in saliva of humans, as well as in a rhesus macaque model of infection [21,22,23,27]. Shedding in tears has also been shown in a mouse model, and the virus has been linked to conjunctivitis and uveitis in human adults and retinal, choroidal, and optic nerve abnormalities in infants born of women infected during pregnancy [24,25,26,28]. Persistent infection and release of viral progeny from DRG neurons, which innervate the genitourinary tract, could contribute to ZIKV shedding in seminal fluid, vaginal secretions, and urine, all of which have been found to contain infectious virus and viral RNA for extended periods of time following infection [29,30]. Although persistent virus in semen is thought to originate from infected testes, recent studies using vasectomized male mice demonstrated that infectious virus and RNA in semen arises from a persistently infected site other than the testes [16]. Furthermore, ejaculate samples from vasectomized humans have remained PCR positive for ZIKV for 77 and 96 days post infection [31,32], validating that ZIKV in genital secretions arises from a site other than the testes in humans, as well. Herpes simplex viruses (HSV1 and HSV2), which also infect and establish lifelong latency in these sensory neurons, cause recurrent orolabial, ocular, and genital lesions with viral shedding in saliva, tears, urine, and genital secretions. Considering we observed no visual evidence of damage or death in ZIKV-infected sensory neurons, our results raise questions as to whether sensory neurons can support long-term or lifelong persistent infection with ZIKV, similar to HSV, to potentially cause recurrent disease or viral shedding months or years after the initial infection. Further studies are needed to address this possibility.

Public concern rests primarily on vertical transmission of ZIKV to fetuses and the consequential congenital syndrome. ZIKV infection during early gestation can lead to miscarriage, stillbirth, intrauterine growth restriction, and microcephaly [33]. Infection of the mother during second or third trimester and prolonged viremia may also contribute to fetal abnormalities [33,34]. Current evidence points toward a transplacental route of infection, which is supported by productive infection of endometrial and placental chorionic villi cells [35,36]. Cytomegalovirus (CMV), a member of the herpesvirus family, is transmitted to fetuses during pregnancy and causes congenital syndrome characterized by ocular impairment, hearing loss, and microcephaly, similar to ZIKV [37]. Sexually transmitted CMV produces high titers of virus in vaginal secretions, which has been associated with an increased miscarriage rate, suggesting CMV in the genital tract could result in adverse pregnancy outcomes [38,39]. Our findings show that sensory neurons innervating the genitourinary tract have the potential to become persistently infected with ZIKV and release infectious viral progeny, providing a reservoir for vaginal shedding of infectious virus. In a mouse model, presence of vaginal ZIKV during pregnancy had a negative impact on fetal growth and led to infection of the fetal brain [40]. Thus, sexual transmission of the virus, and persistent vaginal shedding, may be more likely to produce a negative outcome in the developing fetus compared to mosquito-borne transmission. Although subcutaneous delivery of ZIKV in immunocompetent mice produces only low and transient presence of RNA [41,42], the vaginal route of ZIKV infection supports productive replication in wild-type mice [40], suggesting that the different routes of transmission can produce divergent outcomes of infection in the presence of an intact immune response. Therefore, further studies to identify differences in pathogenesis following mosquito-borne and sexual transmission of the virus, including impact on fetal outcome, are warranted.

**Figure 5:**
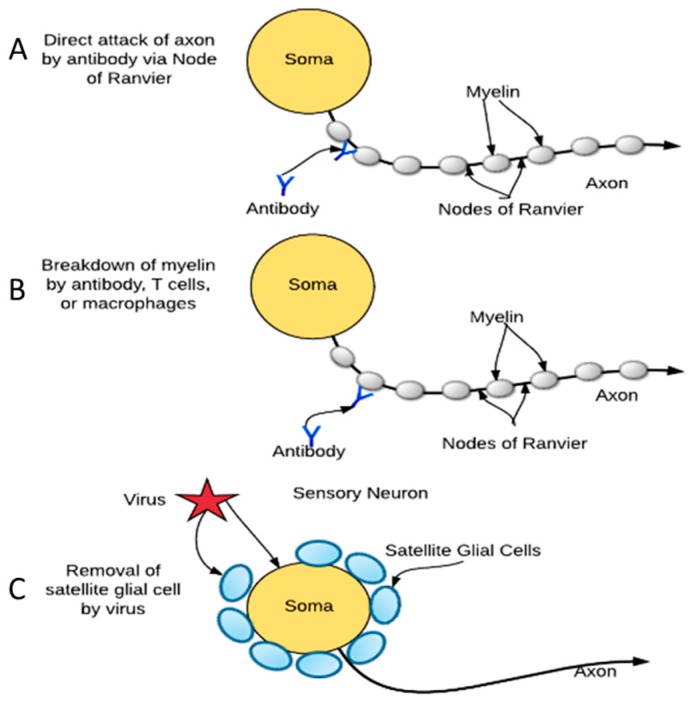
Mechanisms associated with the development of Guillain-Barré Syndrome: (**A**) Attack on axons by cross-reactive antibody and T cells; (**B**) Attack on myelinating cells by cross-reactive antibody and T cells; (**C**) Attack on satellite glial cells surrounding soma of peripheral neurons by viral infection and destruction.

In addition to sensory neuron infection, we found that satellite glial cells, the supporting cells surrounding neurons in peripheral ganglia, are permissive for ZIKV productive infection, which, unlike the neurons, resulted in the rapid death of these cells. Expression of molecules by the satellite glial cells, such as glutamine synthetase (GS), tumor necrosis factor (TNF), and inwardly rectifying potassium ion channels, indicate that they play a role in glutamate uptake and recycling, intercellular signaling, and potassium buffering, an important part of maintaining the resting membrane potential [43]. Given that some pre-synaptic processes synapse on the soma of peripheral neurons, satellite glial cells on these synapses reportedly play an analogous role to astrocytes of the central nervous system (CNS). Taken together, a strong possibility exists that satellite glial cells are an integral part of effective signal transduction along the soma of the neurons of the peripheral ganglia. For the neurons with synapses on the soma, as occurs with sympathetic and parasympathetic neurons, destruction of the satellite glial cells by ZIKV would disrupt synaptic transmission and cause peripheral neuropathies. Infection and destruction of satellite glial cells by ZIKV is an alternative explanation to the putative humoral and T-cell mediated autoimmune dysregulation resulting in physiological and electrophysiological changes that cause Guillain-Barré Syndrome (Figure 5).

In summary, our results show that ZIKV persistently and productively infects adult trigeminal and dorsal root ganglion sensory neurons without apparent damage, but also productively infects supporting satellite glial cells, resulting in their destruction. These findings have implications for persistent viral shedding in semen, vaginal, and other biological secretions, and also provide an alternative mechanism for the development of Guillain-Barré Syndrome following ZIKV infection. Further studies are essential to define differences in pathogenic mechanisms following sexual and mosquito-borne transmission of ZIKV, to determine which route of infection is more likely to contribute to Guillain-Barré Syndrome and produce negative fetal outcomes.

## 4. Materials and Methods

Virus strains: Zika Virus strains MR766 and IbH30696 (BEI Resources) were propagated in Vero cells (ATCC). Titrations of viral stocks were performed in quadruplicate via plaque assay on Vero cells. First passage virus was used for inoculations.

In vitro infection: Sensory trigeminal (TG) and dorsal root ganglia (DRG), and autonomic ciliary (CG) and superior cervical ganglia (SCG) from six-week-old Swiss Webster mice were cultured, as previously described [44]. Briefly, ganglia were collected and dissociated enzymatically and mechanically, followed by neuron enrichment through an Optiprep gradient. Neurons were cultured at a density of 3000 neurons per well on Matrigel-coated 8-well Lab-Tek II chamber slides (ThermoScientific, Waltham MA, USA). Cultures were maintained in Complete Neuro Media, containing Neurobasal A media with 1% penicillin-streptomycin (PS), 2% SM-1 supplement, Glutamax, mitotic inhibitors (prior to infection) and neurotrophic factors nerve growth factor (NGF), neurturin (NTN), and glial cell derived neurotrophic factor (GDNF). Ciliary neurotrophic factor (CNTF) was also included for ciliary ganglia neuronal cultures. The neurons were incubated for three days before inoculation with multiplicity of infection (MOI) of 10 or 30 PFU/cell of IbH30565 or MR766. After a 1 h adsorption period, inoculum was removed by vacuum aspiration and Complete Neuro Media was added (without mitotic inhibitors). The remaining inoculum from infections was back-titrated in duplicate for verification.

Immunofluorescent staining: Human anti-Zika Virus antiserum (KeraFast, Boston, MA, USA; 1:500) was used to identify infected cells, visualized with anti-human secondary antibody conjugated to Alexa Fluor 488 or 594 (Life Technologies, Carlsbad, CA, USA; 1:1000). Stained slides were visualized with Olympus XI71 inverted fluorescent microscope. Infected neurons were counted and presented as the percent of total neurons infected by the viruses. Infections were repeated three to six times, in duplicate, and a minimum of 500 neurons were counted per well in each experiment.

Titration of viral shedding: Medium from infected neurons (100 uL/well) was collected from duplicate wells at designated time points and stored at −80 °C. Inoculum and media from uninfected cells were included as controls. Vero cells were seeded in 24 well plates and grown to 60–70% confluency before infecting in duplicate with 1:10 and 1:100 dilutions of the media samples. After three or four days, the cells were stained and fixed with crystal violet solution. The plates were dried and the number of plaques were counted. Calculations to determine the concentration of plaque forming units (pfu) per ml in the media samples were performed using Microsoft Excel.

Statistics. Percent infection was calculated by counting the number of infected neurons per well and dividing by the total number of neurons per well. Averages and standard error of the mean for percent infection and viral shedding titrations were calculated and graphs were generated in Microsoft Excel. The data were normalized based on the titration of the inoculum for each strain of ZIKV. Student’s *t*-test, two-way ANOVA, and Tukey’s post-hoc analysis were performed to determine significance.
